# Genetic Heterogeneity of Induced Pluripotent Stem Cells: Results from 24 Clones Derived from a Single C57BL/6 Mouse

**DOI:** 10.1371/journal.pone.0120585

**Published:** 2015-03-23

**Authors:** Cheng Li, Jeffery M. Klco, Nichole M. Helton, Daniel R. George, Jacqueline L. Mudd, Christopher A. Miller, Charles Lu, Robert Fulton, Michelle O'Laughlin, Catrina Fronick, Richard K. Wilson, Timothy J. Ley

**Affiliations:** 1 Section of Stem Cell Biology, Division of Oncology, Department of Medicine, Washington University School of Medicine, St. Louis, Missouri, United States of America; 2 Department of Pathology and Immunology, Washington University School of Medicine, St. Louis, Missouri, United States of America; 3 The Genome Institute, Washington University School of Medicine, St. Louis, Missouri, United States of America; University of Kansas Medical Center, UNITED STATES

## Abstract

Induced pluripotent stem cells (iPSCs) have tremendous potential as a tool for disease modeling, drug testing, and other applications. Since the generation of iPSCs “captures” the genetic history of the individual cell that was reprogrammed, iPSC clones (even those derived from the same individual) would be expected to demonstrate genetic heterogeneity. To assess the degree of genetic heterogeneity, and to determine whether some cells are more genetically “fit” for reprogramming, we performed exome sequencing on 24 mouse iPSC clones derived from skin fibroblasts obtained from two different sites of the same 8-week-old C57BL/6J male mouse. While no differences in the coding regions were detected in the two parental fibroblast pools, each clone had a unique genetic signature with a wide range of heterogeneity observed among the individual clones: a total of 383 iPSC variants were validated for the 24 clones (mean 16.0/clone, range 0–45). Since these variants were all present in the vast majority of the cells in each clone (variant allele frequencies of 40–60% for heterozygous variants), they most likely preexisted in the individual cells that were reprogrammed, rather than being acquired during reprogramming or cell passaging. We then tested whether this genetic heterogeneity had functional consequences for hematopoietic development by generating hematopoietic progenitors *in vitro* and enumerating colony forming units (CFUs). While there was a range of hematopoietic potentials among the 24 clones, only one clone failed to differentiate into hematopoietic cells; however, it was able to form a teratoma, proving its pluripotent nature. Further, no specific association was found between the mutational spectrum and the hematopoietic potential of each iPSC clone. These data clearly highlight the genetic heterogeneity present within individual fibroblasts that is captured by iPSC generation, and suggest that most of the changes are random, and functionally benign.

## Introduction

Pluripotent stem cells, such as embryonic stem cells (ESCs), are defined by their ability to self-renew and differentiate into any somatic cell type. In 2006, Yamanaka and colleagues successfully reprogrammed mouse somatic cells into pluripotent stem cells, referred to as induced pluripotent stem cells (iPSCs), by introducing a combination of four transcription factors: *Oct3/4*, *Sox2*, *c-Myc*, and *Klf4*[1]. One year later, both the Yamanaka group and the Thomson group successfully reprogrammed human somatic cells to iPSCs[2,3]. Like ESCs, iPSCs demonstrate unlimited self-renewal in culture, express markers associated with pluripotency (such as alkaline phosphatase and SSEA-1), and can generate teratomas comprised of all 3 germs layers (ectoderm, mesoderm and endoderm) in immunodeficient mice[1].

iPSCs reprogrammed from patient cells can be valuable reagents for studying the pathobiology of specific diseases[4]. However, concerns over the use of iPSCs in translational studies have been raised, including potential associations between human iPSC reprogramming and mutations known to be linked to cancer[5,6]. Previous work from our lab suggested that many of the mutations within iPSCs actually pre-exist in rare cells within the starting cell population[5,7]. Using whole genome sequencing to characterize a small number of mouse iPSCs derived from the same animal, Young *et al*. found a number of shared variants between individual iPSC clones (from one of three experiments) that could be detected in <1 in 500 cells in the parental cell pool[8]. These data suggested that reprogramming, with its associated cell cloning, “captures” the mutational history of each reprogrammed cell, and that some cells within a given starting population may be more fit for reprogramming due to specific background mutations.

Previous studies focused on the genetic heterogeneity of iPSCs have only evaluated a small number of individual clones[9–11], and have been limited in their ability to fully estimate the extent of genetic heterogeneity that results from the reprograming of a pool of somatic cells. In this study, we used exome sequencing to define the genetic heterogeneity of 24 iPSC clones derived from skin fibroblasts taken from two different sites (right and left axilla; 12 clones/site) of the same 8 week old C57BL/6J male mouse.

## Materials and Methods

### Production of murine iPSC clones

The Washington University Animal Studies Committee approved all animal experiments (protocol #20120180). Skin fibroblasts from the right and left axillae (Ax1 and Ax2) of a single healthy 8 week old adult C57BL/6J male mouse were prepared, and iPSC clones were generated as previously described[12]. Briefly, 2.5x10^5^ fibroblasts were seeded on 6-well plates. The next day, the cells were transduced with the OSK-GFP lentivirus (kindly provided by Drs. Joe Sun and Tim Townes) at an MOI of 1:3. After 24 hours of incubation with the virus, the cells were trypsinized and transferred to a 100-mm petri dish with a feeder mouse embryonic fibroblast (MEF) layer and mouse embryonic stem cell (ESC) media containing recombinant LIF (Millipore, Billerica, MA). Cells were grown for 2–3 weeks with daily media changes before individual clones were picked and expanded on MEF feeder layers.

### Pluripotency characterization

GFP and Oct3/4 expression (eBioscience, San Diego, CA) was assessed by flow cytometry after at least 4 weeks of passaging. Alkaline phosphatase staining for all 24 clones was performed using manufacturer’s recommendations (Stemgent, Cambridge, MA). Seven individual iPSC clones (3 with the most robust potential, and 4 with the least robust potential in producing hematopoietic CFUs *in vitro*), were injected into the hind limbs of NSG mice (1x10^7^ cells per mouse, 2 recipients per clone), and teratoma formation was evaluated after 8–12 weeks by histologic examination. Images were captured on the Nikon Eclipse TE300 microscope using the Nikon DXM1200F digital camera.

### *In vitro* hematopoietic differentiation from iPSC

The iPSC hematopoietic differentiation assay is modified from a human iPSC hematopoietic differentiation protocol[13]. Briefly, 1x10^5^ single iPSC or mESCs were seeded in gel-coated 100-mm petri dish with OP9 stromal cells overgrown for 8–10 days in differentiation media containing 10% fetal bovine serum (FBS), 100 μM monothioglycerol (Sigma-Aldrich, St. Louis, MO), and 50 μg/ml ascorbic acid (Sigma-Aldrich, St. Louis, MO). Media was changed daily for 7 days, at which time all the cells in the dish, including OP9s, were collected. Up to 1x10^7^ unsorted cells were stained with the following monoclonal antibodies: Lineage cocktail (B220, CD3ε, Gr-1, Ter119), Kit, Sca-1, CD34, and CD16/32 (FCgamma) (eBioscience, San Diego, CA) and analyzed by flow cytometry. 1x10^5^ unsorted cells were plated into 1.1 ml of methylcellulose media containing Erythropoietin (Epo), SCF, IL-3, and IL-6 (MethoCult GF M3434; Stem Cell Technologies, British Columbia, Canada) in 60-mm petri-dishes in triplicate. Colony numbers were counted after 7–8 days of culture. After dissolving the MethoCult in warm media, cells were stained with the myeloid and erythroid lineage markers CD34, CD11b, Kit, Gr-1, and Ter119 (eBioscience, San Diego, CA) and analyzed by flow cytometry. 1x10^5^ unsorted cells were stained with Wright-Giemsa stain (Sigma-Aldrich, St. Louis, MO) for morphologic examination, both after 7 days of OP9 culture and after another 7 days in MethoCult. Multiple lots of OP9 cells from ATCC and multiple lots and brands of FBS were systematically tested, and neither had a significant influence on hematopoietic differentiation efficiency (S1 Table).

### Illumina library construction and exome sequencing

Genomic DNA from all 24 iPSC clones and the two parental fibroblast lines were fragmented using a Covaris LE220 DNA Sonicator (Covaris, Woburn, MA) within a size range between 100–400 bp using the following settings: volume = 50 μL, temperature = 4°C, duty cycle = 20, intensity = 5, cycle burst = 500, time = 120 seconds. The fragmented samples were transferred from the Covaris plate and dispensed into a 96 well BioRad Cycle plate by the CyBio-SELMA instrument. Small insert dual indexed Illumina paired end libraries were constructed with the KAPA HTP sample prep kit according to the manufacturer's recommendations (KAPA Biosystems, Woburn, MA) on the SciClone instrument according to the manufacturer's recommendations (Perkin Elmer, Waltham, MA). Dual indexed adaptors were incorporated during ligation; the same 8bp index sequence is embedded within both arms of the library adaptor. Libraries were enriched with a single PCR reaction for 8 cycles. The final size selection of the library was achieved by a single AMPure XP paramagnetic beads (Agencourt, Beckman Coulter Genomics, Beverly, MA) cleanup targeting a final library size of 300–500bp. The libraries underwent a qualitative (final size distribution) and quantitative assay using the HT DNA Hi Sens Dual Protocol Assay with the HT DNA 1K/12K chip on the LabChip GX instrument (Perkin Elmer, Waltham, MA). Twenty-six libraries (from the 24 iPSC clones and the two parental fibroblast pools), at 192 ng per library, were pooled pre-capture on the Ep5075 platform, captured (see Exome capture, and validation capture), and sequenced on an Illumina HiSeq 2000 using 100 bp paired-end reads. Exome sequencing coverage for the 24 iPSC clones and the fibroblast preparations from which they were derived are included in Table 1.

**Table 1:** Whole exome sequencing coverage.

Sample	NimbleGen, mean	NimbleGen, median	Agilent, Mean	Agilent, Median
Ax1-2	50.97	39.91	71.24	58.69
Ax1-3	50.72	37.85	67.02	55.19
Ax1-5	53.99	42.99	75.18	60.49
Ax1-7	47.38	35.97	65.11	53.14
Ax1-8	49.87	38.76	68.97	57.88
Ax1-10	42.04	31.79	59.70	48.90
Ax1-11	50.68	38.94	67.58	55.65
Ax1-14	49.18	37.68	66.41	55.68
Ax1-16	71.62	55.49	102.50	83.39
Ax1-18	52.35	40.72	73.54	59.80
Ax1-23	46.82	35.28	63.74	52.18
Ax1-35	65.58	50.83	90.71	74.75
Ax1 parental fibroblast	39.76	31.21	51.00	42.39
Ax2-4	51.18	40.33	72.68	60.48
Ax2-6	53.40	42.19	75.34	61.58
Ax2-11	51.03	39.96	73.56	59.24
Ax2-16	47.93	36.47	66.50	54.69
Ax2-20	44.55	33.72	62.85	51.43
Ax2-24	47.22	36.68	68.59	56.31
Ax2-26	54.30	42.27	73.54	62.44
Ax2-27	48.11	36.59	64.76	53.11
Ax2-30	43.88	33.61	60.87	49.81
Ax2-34	41.62	32.17	60.06	49.29
Ax2-39	56.74	44.10	80.00	64.48
Ax2-48	45.28	34.37	61.48	50.22
Ax2 parental fibroblast	67.05	53.26	91.18	75.36
Mean	50.89	39.35	70.54	57.95

The sequencing data for all 26 exomes has been deposited in the Short Read Archive (http://trace.ncbi.nlm.nih.gov/Traces/study/?acc=SRP051818); the SRA study accession number is SRP051818.

### Variant detection pipeline

Sequence data was aligned to mouse reference sequence mm9 (with the OSK vector sequence added) using bwa version 0.5.9[14] (params:-t 4-q 5::). Bam files were deduplicated using picard version 1.46.

Single Nucleotide Variants (SNVs) were detected using the union of three callers: 1) samtools version r963[15] (params:-A-B) intersected with Somatic Sniper version 1.0.2[16] (params:-F vcf-q 1-Q 15) and processed through false-positive filter v1 (params:—bam-readcount-version 0.4—bam-readcount-min-base-quality 15—min-mapping-quality 40—min-somatic-score 40) 2) VarScan version 2.2.6[17] filtered by varscan-high-confidence filter version v1 and processed through false-positive filter v1 (params:—bam-readcount-version 0.4—bam-readcount-min-base-quality 15—min-mapping-quality 40—min-somatic-score 40), and 3) Strelka version 0.4.6.2[18] (params: isSkipDepthFilters = 1).

Indels were detected using the union of 4 callers: 1) GATK somatic-indel version 5336[19] filtered by false-indel version v1 (params:—bam-readcount-version 0.4—bam-readcount-min-base-quality 15), 2) pindel version 0.5[20] filtered with pindel false-positive and vaf filters (params:—variant-freq-cutoff = 0.08), 3) VarScan version 2.2.6[17] [filtered by varscan-high-confidence-indel version v1 then false-indel version v1 (params:—bam-readcount-version 0.4—bam-readcount-min-base-quality 15), and 3) Strelka version 0.4.6.2[18] (params: isSkipDepthFilters = 1).

Variants were filtered to remove non-homozygous or heterozygous sites using an R script (https://github.com/genome/gms-core/blob/f00200864a9d0b87e6b6257c5e6bcadab4e6f685/lib/perl/Genome/Model/Tools/Analysis/RemoveContaminatingVariants.R)

Viral integration sites were detected using Breakdancer version 1.4.1[21] and when possible, assembled using TIGRA-SV (http://gmt.genome.wustl.edu/tigra-sv). Integration “hotspots” were defined as 50 kbp regions containing integration events in more than one sample.

### Exome capture and capture validation

Two library pools were made for exome capture, each containing all 26 libraries and a total input of ∼5ug into capture. One pool was captured using the Agilent SureSelect Mouse All Exon Library Kit according to manufacturer's recommendations with these exceptions:

1. 5 μg Mouse Cot DNA and 1mM library adapter blockers were added to the hybridization reaction.
2. Each sample was amplified in the PCR using 20μl of enriched ssDNA library fragments, KAPA HotStart Polymerase, and 200nM each forward primer and reverse primer.

The other pool was captured using the Nimblegen SeqCap EZ Library reagent with the same exceptions. Both products have a probe space of ∼50Mb. The final concentration of each capture pool was verified through qPCR utilizing the KAPA Library Quantification Kit—Illumina/LightCycler 480 kit according to the manufacturer's protocol (Kapa Biosystems, Woburn, MA) to produce cluster counts appropriate for the Illumina HiSeq2000 platform. Each capture pool was loaded across 5 lanes of the HiSeq2000 version 3 flow cell according to the manufacturer's recommendations (Illumina, San Diego, CA). 2 X 101bp read pairs were generated for each sample, yielding approximately 6–7 Gb of data per sample.

For the validation array, genomic DNA of all 24 miPSC clones was isolated from sorted GFP positive iPSCs to minimize MEF contamination. The custom capture reagent (NimbleGen) contained all predicted somatic mutations from all 24 iPSC clones, as well as 25 probes that tiled the OSK sequence to map the integration sites. Capture was performed as described above for the NimbleGen exome reagent.

## Results

### Genetic heterogeneity among miPSC clones derived from the same parental fibroblasts

**Fig 1:**
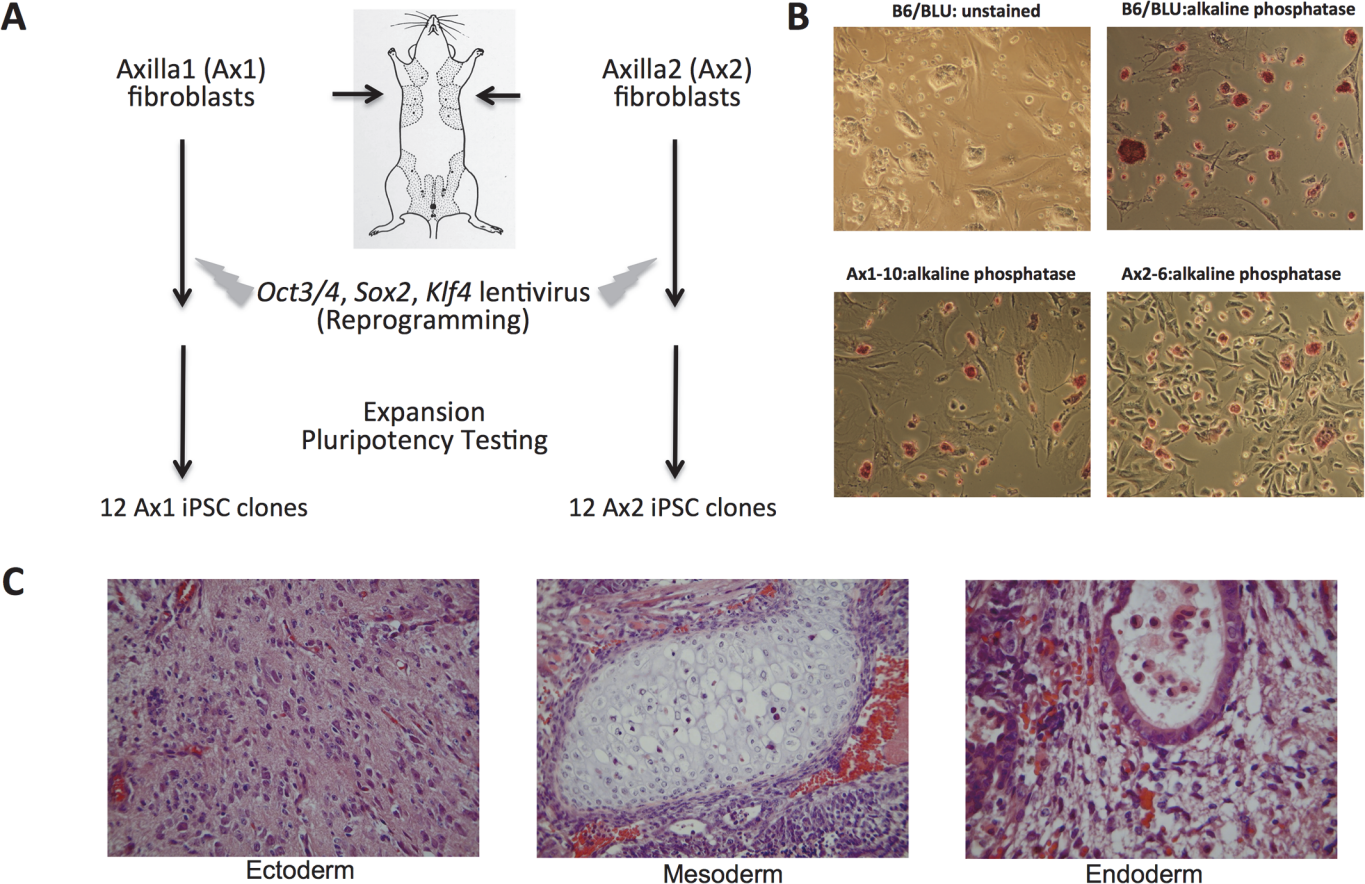
Generation of iPSC clones from a single mouse C57BL/6 male mouse. **A.** Schematic of experimental approach (see Material and Methods for full protocol). **B.** Representative bright-field images (left) and alkaline-phosphastase stains (right) of B6/BLU ESCs (top) and a representative iPSC (bottom, Ax1-10). All images at 100x magnification. **C.** Images from the teratoma derived from Ax1-10 demonstrating ectoderm (neural tissue), mesoderm (cartilage) and endoderm (ciliated respiratory epithelium).

24 miPSC clones were generated from the same adult male C57BL/6J mouse using a polycistronic lentivirus containing cDNAs encoding *OCT3/4*, *SOX2*, *KLF4* (OSK)[12], and an IRES-*GFP* cassette to mark stably transduced cells. These clones were generated at the same time from two independent fibroblast pools from the right and left axillae; 12 clones were expanded from each pool (Fig. 1A). All clones were GFP positive, and expressed Oct3/4 and alkaline phosphatase (Fig. 1B and S2 Table). Seven of the 24 iPSC clones (Ax1-10, Ax1-18, Ax1-35, Ax2-26, Ax2-34, Ax2-39, and Ax2-48) were evaluated for pluripotency by injection into immunodeficient mice; all clones produced teratomas containing tissues derived from the endoderm, mesoderm and ectoderm (Fig. 1C).

Exome sequencing on all 24 iPSC clones, as well as the two parental fibroblast pools from which they were derived, was then performed to comprehensively define the genetic heterogeneity within iPSCs generated from the same mouse at the same time. Each sample was sequenced with two different exome reagents (Agilent SureSelect Mouse Exon, mean coverage 70.5X, and Nimblegen SeqCap EZ, mean coverage 50.9X) to ensure adequate coverage across the exome space (Table 1). Using genomic DNA collected from iPSCs purified by flow cytometry (GFP-positive), all putative variants from both platforms were then validated using a liquid phase custom capture array and deep digital sequencing (mean coverage >600X), which also allowed us to accurately determine the variant allele frequency (VAF) of each variant (Table 2 and S3 Table).

**Table 2:** Validation array coverage.

Sample	Mean Coverage	Median Coverage
Ax1-2	571.98	431.46
Ax1-3	800.73	606.67
Ax1-5	618.36	465.09
Ax1-7	853.38	652.16
Ax1-8	532.74	401.38
Ax1-10	495.42	371.31
Ax1-11	946.35	727.55
Ax1-14	928.85	703.41
Ax1-16	597.40	452.26
Ax1-18	480.24	348.80
Ax1-23	470.90	350.81
Ax1-35	404.18	303.20
Ax1 parental fibroblast	417.30	303.40
Ax2-4	665.04	500.78
Ax2-6	538.68	403.43
Ax2-11	447.50	335.88
Ax2-16	450.58	338.16
Ax2-20	694.01	532.81
Ax2-24	562.92	422.89
Ax2-26	530.64	396.95
Ax2-27	549.81	414.58
Ax2-30	916.81	695.59
Ax2-34	556.11	417.81
Ax2-39	531.33	396.36
Ax2-48	533.86	398.94
Ax2 parental fibroblast	621.84	454.89
Mean	604.50	454.87

**Fig 2:**
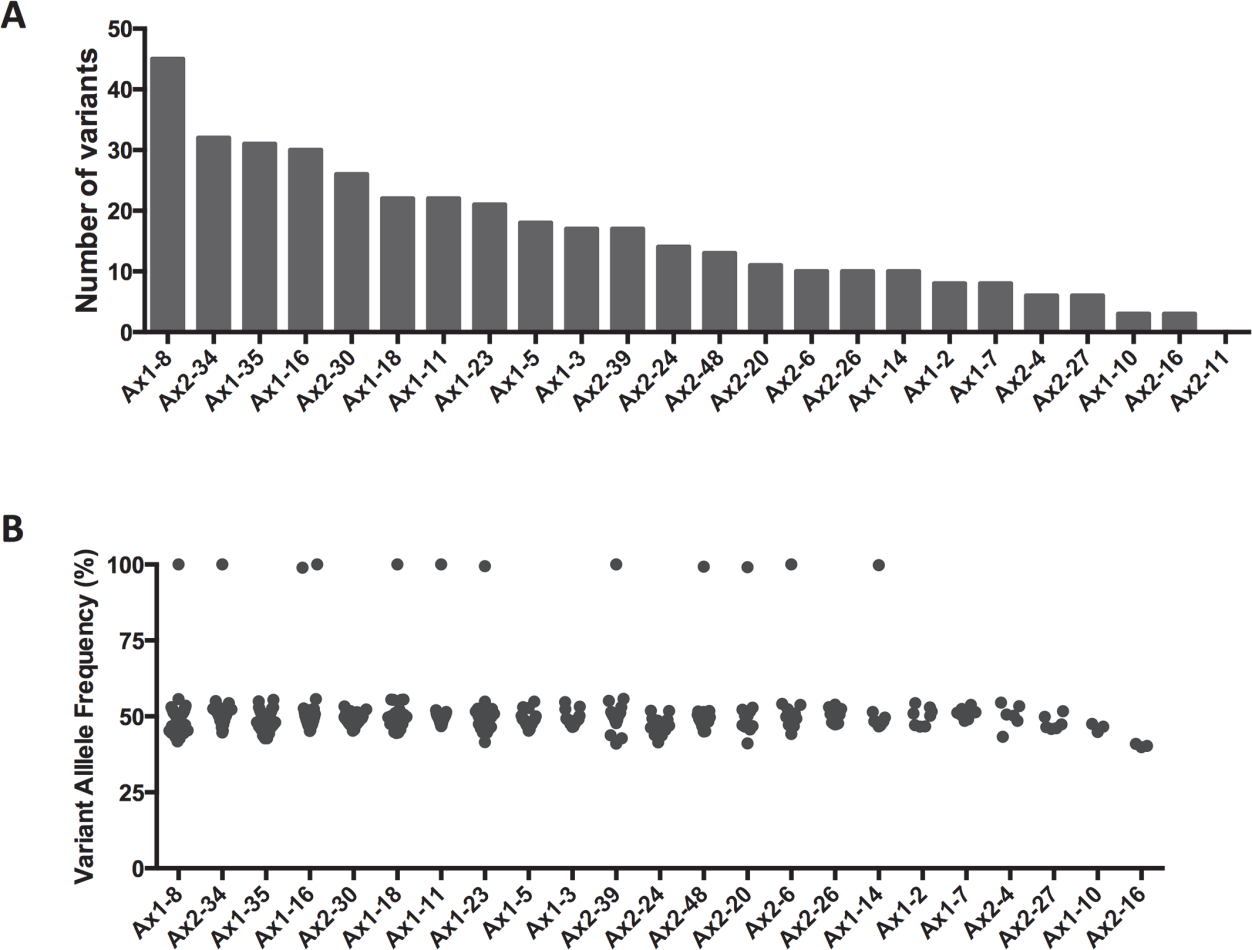
Sequencing of iPSC clones. **A.** Number of variants identified per iPSC clone. **B.** Variant allele frequencies of all validated mutations for each clone. Samples are ranked by the number of variants in decreasing order. No mutations were identified in Ax2-11, thus it is not listed in panel B.

We sought to specifically identify variants present in the single cell that was reprogrammed by the OSK virus, rather than detecting rare variants that arose during the reprogramming process or the post-reprogramming expansion and culture. Thus, we focused exclusively on variants present in nearly all of the cells of an iPSC clone (heterozygous variants at a variant allele frequency [VAF] of 40–60%, or homozygous variants with VAFs >80%, with a coverage threshold of >100x). Using these criteria, no differences were detected between the two parental fibroblast pools from the same mouse, as expected. In contrast, when comparing the iPSCs to their parental fibroblasts, a total of 383 iPSC variants were detected in the 24 clones (mean 16.0, range 0–45) (Fig. 2A-B). The majority of these variants (343 of the 383 total variants) had no supporting reads at a mean coverage depth of 320x in the Ax1 parental fibroblasts, and 462x in the Ax2 parental fibroblasts, while the remaining 40 variants could be detected at very low VAFs in the parental fibroblasts (S3 and S4 Tables). In total, the mean VAF of the iPSC variants in the parental fibroblasts was 0.08% (median 0.00%, range: 0.00–4.69%).

Only 1 variant was shared among different clones: 4 clones (Ax1-5, Ax1-23, Ax2-24 and Ax2-34) harbored the identical missense substitution in *Dcbld1* (Chr10, 52024482 A>G) resulting in an I216M amino acid change. Analysis of the sequencing data from the parental fibroblasts detected this variant at a VAF of 4.69% in Ax1, and 3.11% in Ax2 fibroblasts, suggesting that a small fraction of cells within each independent skin sample (between 5 and 10% of cells) contained this mutation; it probably represents mosaicism within the skin of this animal. This variant was present in the majority of the cells in the four iPS clones (VAF range 48.02–52.13%), clearly demonstrating how preexisting mutations in parental cells are captured by iPSC reprogramming. To determine whether this mutation improved fitness for reprogramming, we analyzed a total of 96 iPSC clones from the skin fibroblasts of this mouse, and found the variant in a total of 9 clones (9.4%). From the combined VAF of the primary skin samples (32 out of 855 total reads, VAF = 3.74%), we estimate that ∼7.5% of the skin cells contained this heterozygous variant. In total, these observations suggest that the *Dcbld1* variant had little if any effect on the fitness of fibroblasts for reprogramming.

An average of 3 OSK lentiviral integration sites (range 1–8) were identified in the 24 miPSC clones (S5 Table). All of the 24 clones had a unique set of integration events, establishing their unique clonal identities. Ax2-11 and Ax2-30 shared one identical integration site on the X chromosome (S6 Table), yet Ax2-30 had 8 total integration sites compared to only 2 for Ax2-11. These two clones also did not have any common variants when compared to the Ax2 fibroblast pool. In fact, we did not identify any coding variants in Ax2-11. Overall, there was no association between the number of integration sites and the number of variants identified by exome sequencing (data not shown). No insertion events were identified in genes known to be important in ESC/iPSC function or hematopoietic development (data not shown). Several integration “hotspots” were identified: Chr2: 98502394–98507455 (14 clones from both Ax1 and Ax2), Chr9: 3000297–3034834 (15 clones from both Ax1 and Ax2), and ChrX: 100516717–100525474 (5 clones from both Ax1 and Ax2). Of these, only the hotspot on Chromosome 2 has previously been reported [22] (S7 Table).

### Functional heterogeneity among miPSC clones derived from the same parental fibroblasts

**Fig 3:**
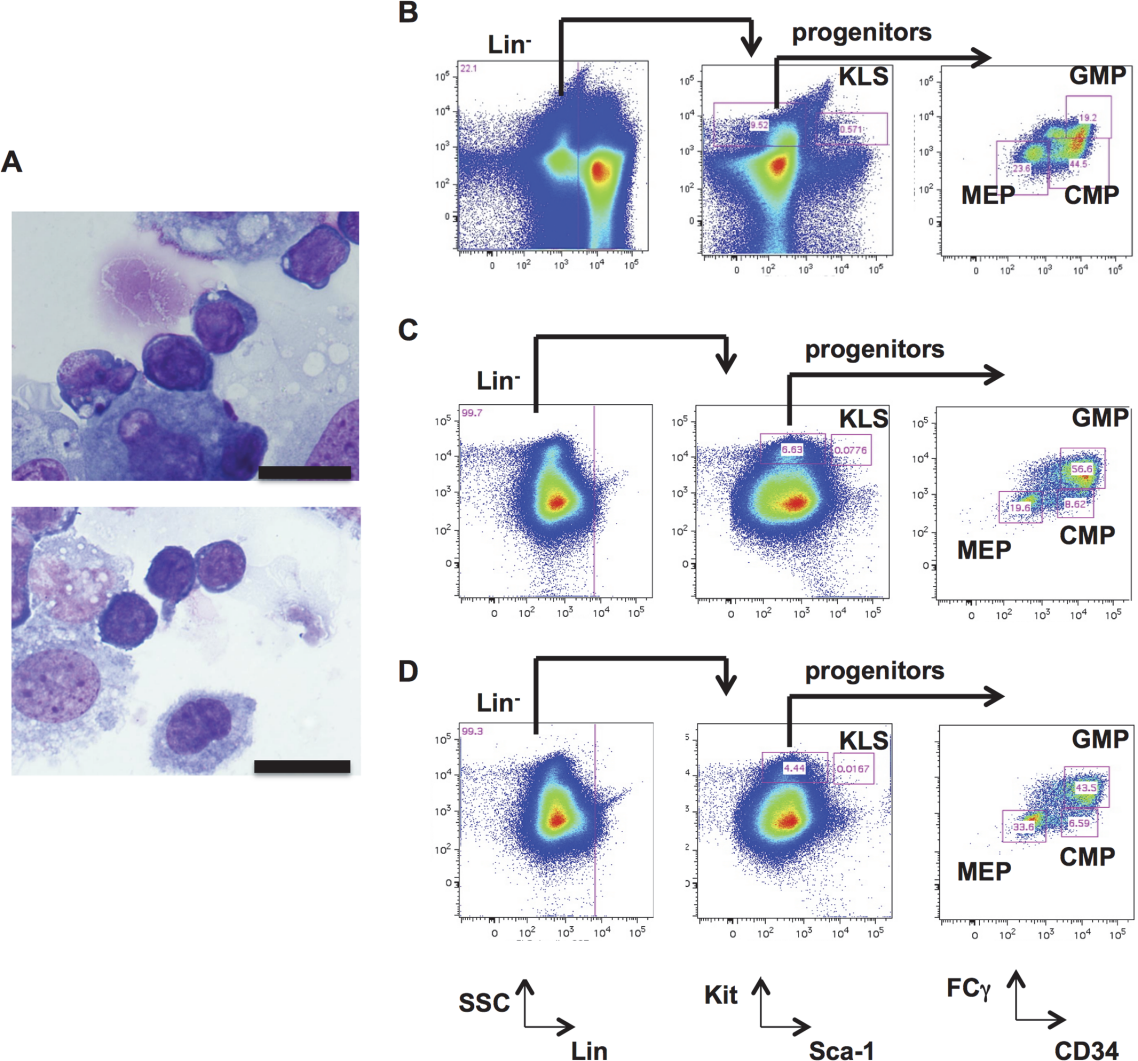
Hematopoietic differentiation from murine ESCs. **A.** Morphology of wild type ESC-derived cells after 7 days of OP9 coculture (unsorted) by Wright-Giemsa staining. A scale bar of 20 μm is shown. (B-D). Immunophenotyping of hematopoietic progenitor cells from wild-type mouse bone marrow cells (panel B), murine ESCs after 7 days of OP9 coculture (panel C), and iPSC clone Ax1-14 after 7 days of OP9 coculture (panel D). Lineage^−^ (Lin^−^), KLS (Lin^−^Kit^+^Sca-1^+^), progenitors (Lin^−^Kit^+^Sca-1^−^), CMPs (Lin^−^Kit^+^Sca-1^−^CD34^+^FCγ^−^), GMPs (Lin^−^Kit^+^Sca-1^−^CD34^+^FCγ^+^), and MEPs (Lin^−^Kit^+^Sca-1^−^CD34^−^FCγ-).

**Fig 4:**
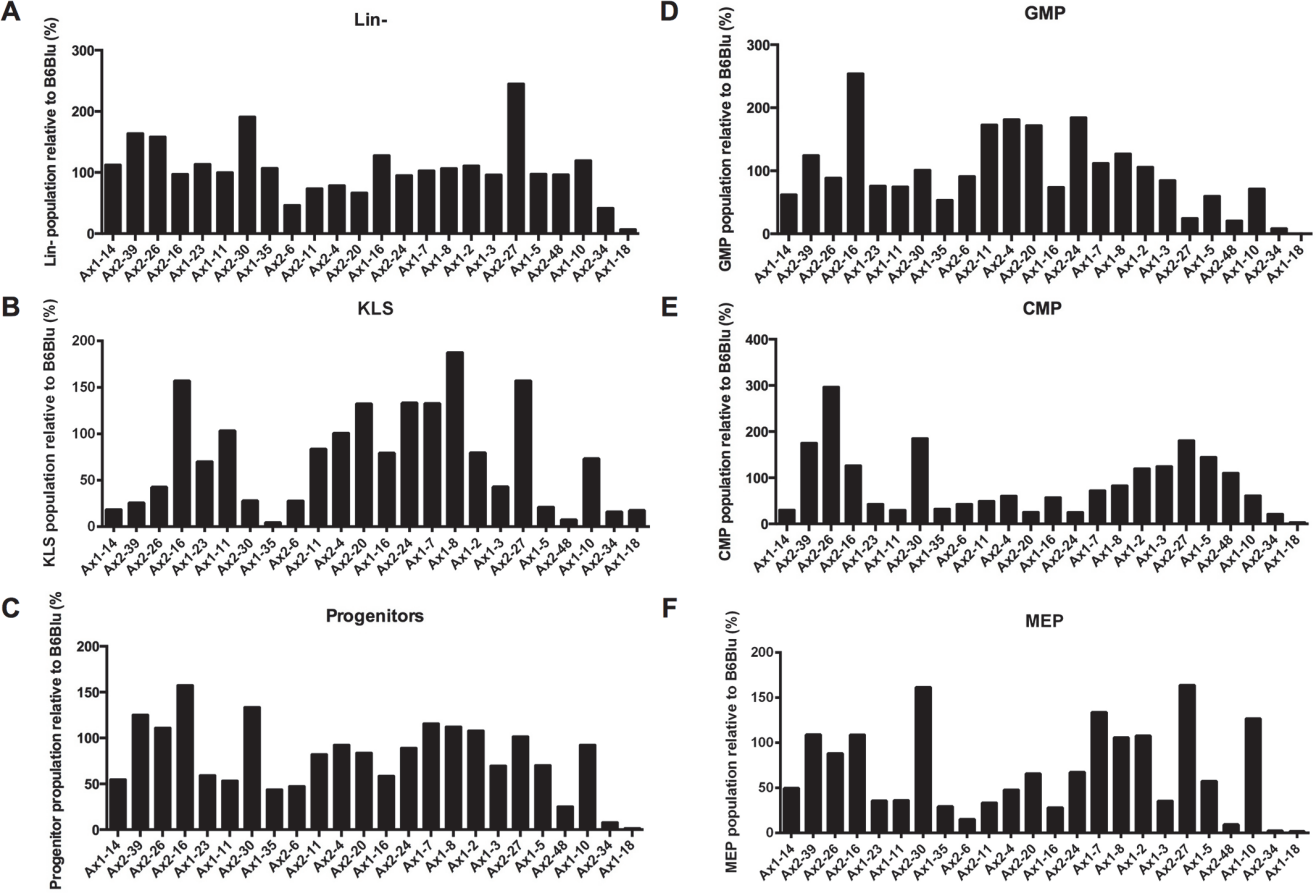
Comparison of hematopoietic potential of iPSCs to mouse ESCs. Fractions of Lin^−^ cells (A), KLS cells (B), Kit+Lin-Sca-1- Progenitors (C), GMPs (D), CMPs (E), and MEPs (F) from iPSCs relative to mouse ESCs after 7 days of OP9 coculture (unsorted).

To determine whether functional heterogeneity existed among the 24 iPSC clones derived from the same parental cells, we modified a protocol for hiPSC hematopoietic differentiation to induce the production of murine hematopoietic stem/progenitor cells (HSPCs) from ESCs and iPSCs. As described in Methods and Materials, after co-culture on OP9 stromal cells for one week, control wild type mESC lines derived from C57BL/6 mice (B6/BLU or B6/GFP) consistently differentiated into hematopoietic progenitors, as determined by morphologic examination (Fig. 3A). These cells also had immunophenotypic characteristics of KLS cells (Kit^+^Lin^−^Sca-1^+^), common myeloid progenitors (CMPs), granulocyte-macrophage progenitors (GMPs), and megakaryocyte-erythroid progenitors (MEPs) (Fig. 3B-D). The iPSC clones were capable of differentiating into KLS cells, GMPs, CMPs, and MEPs with variable efficiencies compared to ESCs. Only Ax1-18 consistently failed to produce immunophenotypically-defined hematopoietic progenitors (Fig. 4).

**Fig 5:**
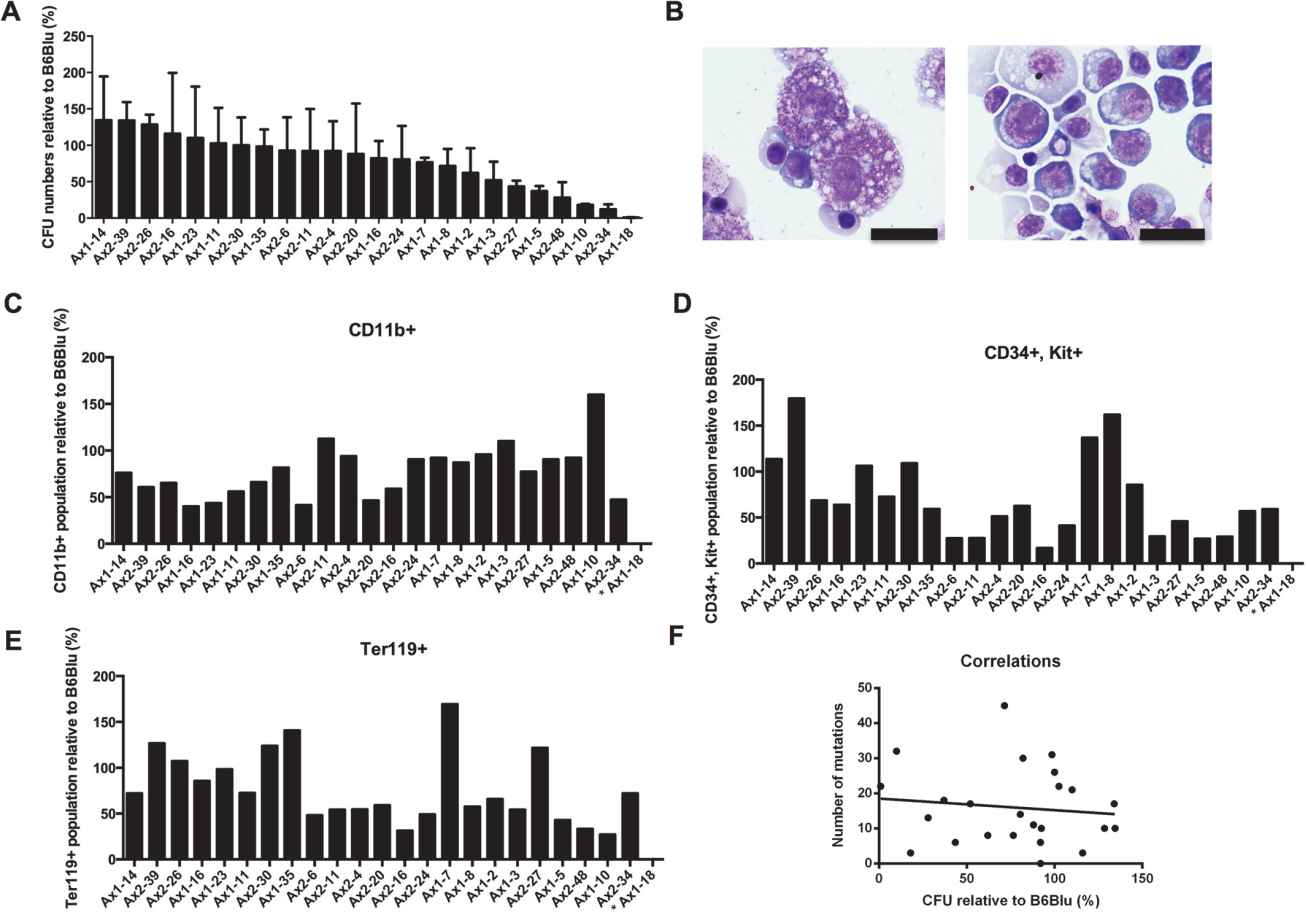
Hematopoietic differentiation potential of the 24 iPSC clones. 100,000 cells from OP9 cocultured mESCs (B6/BLU) or iPSCs were plated in methylcellulose media containing hematopoietic cytokines (SCF, IL-3, IL-6, and Epo). **A.** CFUs were counted after 7 additional days of culture. The relative number of CFUs per 100,000 cells plated from Day7 iPSC-derived progenitors vs. Day7 ESC (B6/BLU)-derived progenitors are shown. iPSC clones are ranked from the highest to the lowest average of two independent experiments. Error bars represent the means +/− one standard deviation. **B.** Morphology of day 7 OP9 cocultured ESC-derived cells after 7–8 days of additional culture in MethoCult media containing hematopoietic cytokines (SCF, IL-3, IL-6, and Epo). A scale bar of 20 μm is shown. **(C-E)**. Fractions of CD11b^+^ (**C**), CD34^+^Kit^+^ (**D**), and Ter119^+^ (**E**) cells obtained after 7 days of methylcellulose culture containing hematopoietic cytokines (SCF, IL-3, IL-6, and Epo), comparing iPSC-derived progenitors relative to ESC-derived progenitors, in the same order as panel A. **F.** Lack of correlation between the number of mutations and the hematopoietic differentiation potential of the iPSC clones (r^2^ = 0.0006065).

To assess the hematopoietic potential of these immunophenotypically-defined hematopoietic progenitors, cells grown for 1 week on OP9 feeder cultures were then grown in methylcellulose with hematopoietic cytokines (SCF, IL-3, IL-6, and Epo) for 7 days, and colony forming units (CFUs) were enumerated. In two independent experiments, the 24 miPSC clones exhibited variable but reproducible potentials in their ability to produce functional hematopoietic progenitor cells (Fig. 5A). Further, erythrocytes and mast cells were readily identified by morphologic examination in the methylcellulose cultures (Fig. 5B); cells expressing CD34, Kit, Ter119, and CD11b were also detected at variable levels (Fig. 5C-E). Consistent with the results from the progenitor studies above, Ax1-18 was incapable of forming colonies in cytokine-supplemented methylcellulose. There was no correlation between the hematopoietic differentiation potential of the iPSC clones and the number of mutations (r^2^ = 0.01339) (Fig. 5F) or the number of integration sites (data not shown).

## Discussion

The generation of iPSCs starts with a single somatic cell. All of the cells in the resulting iPSC clone would therefore be expected to harbor the somatic alterations present in that individual founding cell, in essence “capturing” the mutational history of that cell. The genetic heterogeneity that results from this cloning of individual cells has been addressed in some studies using small numbers of iPSC clones. To more comprehensively address the degree of genetic heterogeneity that can result from iPSC generation, we characterized the exomic mutations in 24 iPSCs generated from the same C57BL/6 male mouse, using two independent preparations of skin fibroblasts. We found that there was a wide range in the number of mutations in the exomes of individual iPSC clones when compared to the pool of fibroblasts that served as the cell source for iPSC production, highlighting the genetic heterogeneity inherent in iPSC generation.

In this study, we limited our analysis to genetic alterations present in nearly all the cells of the iPSC clone (variants with VAFs of ∼50%), since these would most likely have been present in the individual fibroblasts that were reprogrammed. In contrast, mutations present in a subset of the cells of the iPSC clone would most likely arise during cell expansion, and were therefore excluded from this analysis. This approach was chosen based on our previous study, which strongly suggested that most variants present in an individual iPSC were carried forward from the reprogrammed cell [8]. Most of the variants detected in this study had few if any variant reads in the parental fibroblasts. We favor the hypothesis that these variants were pre-existing in the rare cells that were reprogrammed, and were below our limit of detection by sequencing. However, we cannot exclude the possibility that they occurred during the initial stages of reprogramming. Another possibility is that these mutations arose during expansion, and that specific mutations provided clones with a strong proliferative advantage; if this were the case, we would expect to see recurring patterns of mutations in genes that are known to provide a proliferative signal (e.g. activating mutations in Ras, tyrosine kinase receptors, etc.). However, no mutations of this class were identified in any of the clones. For some variants, such as the A>G transition in *Dcbld1*, our results convincingly demonstrate that these variants were present in a subset of cells in the fibroblast pools used for iPSC generation, and that the mutation likely provided no fitness advantage for reprogramming.

Functional heterogeneity in iPSCs has been documented by several other studies [9,23–25], but all have examined only a small number of clones. Our experimental design allowed us to test the extent of functional heterogeneity among a large set of iPSCs and to determine if there was an association with genetic alterations. We did observe a range in the ability of individual iPSCs to differentiate into hematopoietic cells. Other studies have likewise demonstrated variable potentials of iPSCs derived from a common source to differentiate into a specific lineage, such as neurons [26,27], hematopoietic progenitors [9,27], or hepatocytes [24]. In this study, only one of 24 clones was unable generate hematopoietic progenitors. This clone, Ax1-18, had a similar number of exomic mutations (n = 22) to other clones in the set, and none were in genes that are currently known to be important for hematopoietic differentiation or development (S3 Table). In addition, this clone was capable of forming teratomas *in vivo*, confirming its pluripotency. It is possible that epigenetic differences captured in the reprogrammed cell may have influenced its functional properties [26–28].

While our data clearly show there is little association between functional and genetic heterogeneity in this group of 24 iPSCs, it is not yet clear how genetic heterogeneity will impact disease-specific, patient-derived iPSCs. This may especially be true in iPSCs derived from primary, patient-derived tumor samples, which are inherently heterogeneous in their genetic makeup, containing not only a set of passenger mutations acquired over the life of the transformed cell, but also mutations that influence cell growth and differentiation. Indeed, we have shown that primary AML samples consist of different populations of cells with mutations that can confer unique functional properties [29]. Considering these data, it would not be surprising if the functional heterogeneity of iPSCs is more prominent when derived from tumor samples—or even from patients with cancer predisposition syndromes, which may have altered background mutation rates.

In summary, we characterized the mutational landscape of 24 iPSC clones derived from the same mouse. While we found a substantial amount of genetic heterogeneity, only one of the clones was incapable of hematopoietic differentiation, and there was no correlation between the number of mutations and the ability to generate functional hematopoietic progenitors. Future studies are clearly warranted with different initial cell sources for iPSC generation (e.g. bone marrow/peripheral blood mononuclear cells and patient-derived primary samples), and different lineage differentiation strategies. Regardless, these data provide a large database and reference material for future iPSC experiments focused on heterogeneity among iPSC clones.

## Supporting Information

S1 Table OP9 and FBS lot compatibility with the hematopoietic differentiation assay, based on number of CFUs produced by B6 ESC-derived hematopoietic progenitors. (DOCX)

S2 Table GFP and Oct3/4 expression miPSC clones as measured by flow cytometry. (DOCX)

S3 Table Validated variants in all 24 miPSC clones. (XLSX)

S4 Table Variants detected at low VAFs in the parental fibroblasts. (XLSX)

S5 Table OSK lentiviral integration sites. (DOCX)

S6 Table Common OSK lentiviral integration sites. (DOCX)

S7 Table Integration “hotspots”. (DOCX)
