# Supplementary material for: Genetic Heterogeneity of Induced Pluripotent Stem Cells: Results from 24 Clones Derived from a Single C57BL/6 Mouse
Source: PLoS One. 2015 Mar 23;10(3):e0120585. doi: 10.1371/journal.pone.0120585 (PMC4370741; doi:10.1371/journal.pone.0120585)
Supplement: S1 Table — (DOCX) [file pone.0120585.s001.docx]

**Table S1.** OP9 and FBS lot compatibility (Based on number of CFUs produced by B6 ESC-derived hematopoietic progenitors) with the hematopoietic differentiation assay

| **OP9 ATCC Lot#** | **FBS Brand & Lot#** | **Compatibility with hematopoietic differentiation assay** |
| --- | --- | --- |
| Lot # 58105522 | FisherBrand – Research Grade Serum – Cat# 03-600-510 (Lot# FB-004) | ✔ |
| Lot # 58105522 | Hyclone – FBS Characterized – Cat# SH30071 (Lot# AXF42326) | ✔ |
| Lot # 58105522 | Hyclone – FBS Characterized – Cat # SH30071 (Lot# AXE41320) | ✔ |
| Lot # 58105522 | Hyclone – FBS Characterized – Cat# SH30071 (Lot# AWC99942) | ✔ |
| Lot # 60484552 | Hyclone – FBS Characterized – Cat# SH30071 (Lot# AWC99942) | ✔ |
| Lot # 60053041 | Hyclone – FBS Characterized – Cat# SH30071 (Lot# AWC99942) | ✔ |
