# Supplementary material for: Genetic Heterogeneity of Induced Pluripotent Stem Cells: Results from 24 Clones Derived from a Single C57BL/6 Mouse
Source: PLoS One. 2015 Mar 23;10(3):e0120585. doi: 10.1371/journal.pone.0120585 (PMC4370741; doi:10.1371/journal.pone.0120585)
Supplement: S2 Table — (DOCX) [file pone.0120585.s002.docx]

**Table S2.** GFP and Oct3/4 expression measured by flow cytometry. Values represent percentage of live cells with expression. Alkaline phosphatase positivity measured by visual inspection of clones following staining.

| **Clone** | **GFP** | **Oct3/4** | **Alkaline Phosphatase** |
| --- | --- | --- | --- |
| Ax1-2 | 96.29% | 99.40% | + |
| Ax1-3 | 82.35% | 94.00% | + |
| Ax1-5 | 86.86% | 99.95% | + |
| Ax1-7 | 83.72% | 99.70% | + |
| Ax1-8 | 88.81% | 99.77% | + |
| Ax1-10 | 91.29% | 91.00% | + |
| Ax1-11 | 76.23% | 99.60% | + |
| Ax1-14 | 82.73% | 99.48% | + |
| Ax1-16 | 74.90% | 99.91% | + |
| Ax1-18 | 94.53% | 99.91% | + |
| Ax1-23 | 93.77% | 99.92% | + |
| Ax1-35 | 90.93% | 99.96% | + |
| Ax2-4 | 79.28% | 98.30% | + |
| Ax2-6 | 91.59% | 98.96% | + |
| Ax2-11 | 88.64% | 95.20% | + |
| Ax2-16 | 81.58% | 98.20% | + |
| Ax2-20 | 82.96% | 97.43% | + |
| Ax2-24 | 88.96% | 98.10% | + |
| Ax2-26 | 41.31% | 98.43% | + |
| Ax2-27 | 93.48% | 98.86% | + |
| Ax2-30 | 95.32% | 94.78% | + |
| Ax2-34 | 89.43% | 98.94% | + |
| Ax2-39 | 70.65% | 91.24% | + |
| Ax2-48 | 91.72% | 97.31% | + |
