# Supplementary material for: Genetic Heterogeneity of Induced Pluripotent Stem Cells: Results from 24 Clones Derived from a Single C57BL/6 Mouse
Source: PLoS One. 2015 Mar 23;10(3):e0120585. doi: 10.1371/journal.pone.0120585 (PMC4370741; doi:10.1371/journal.pone.0120585)
Supplement: S5 Table — (DOCX) [file pone.0120585.s005.docx]

**Table S5.** OSK lentiviral integration sites.

| **Clone** | **Chromosome** | **Start** | **End** | **Supporting Reads** | **Gene location** |
| --- | --- | --- | --- | --- | --- |
| Ax1-2 | 2 | 98506736 | 98507278 | 4 |  |
| Ax1-2 | 18 | 42403351 | 42403680 | 7 |  |
| Ax1-3 | 2 | 5804912 | 5805384 | 15 | Cdc123 |
| Ax1-3 | 2 | 98502410 | 98507353 | 9 |  |
| Ax1-3 | 4 | 130274243 | 130274619 | 14 | Serinc2 |
| Ax1-3 | 9 | 3002047 | 3007568 | 3 |  |
| Ax1-3 | 9 | 3024074 | 3027195 | 6 |  |
| Ax1-3 | 17 | 25063321 | 25063633 | 12 | Ift140 and Tmem204 |
| Ax1-5 | 2 | 98502397 | 98507406 | 40 |  |
| Ax1-5 | 4 | 129341285 | 129341600 | 3 | Zbtb8os |
| Ax1-5 | 9 | 3000297 | 3017965 | 25 |  |
| Ax1-5 | 9 | 3020843 | 3032855 | 18 |  |
| Ax1-5 | 9 | 56271726 | 56272024 | 16 | Peak1 |
| Ax1-5 | 12 | 3109872 | 3109978 | 8 |  |
| Ax1-7 | 2 | 98506703 | 98507261 | 3 |  |
| Ax1-7 | 4 | 98180096 | 98180353 | 10 |  |
| Ax1-8 | 9 | 71779801 | 71780302 | 12 |  |
| Ax1-8 | 14 | 8395160 | 8395240 | 4 |  |
| Ax1-10 | 1 | 164653171 | 164653431 | 9 |  |
| Ax1-10 | 5 | 51426546 | 51426987 | 4 |  |
| Ax1-10 | 13 | 55365658 | 55366178 | 8 |  |
| Ax1-11 | 2 | 98502678 | 98507455 | 14 |  |
| Ax1-11 | 8 | 35736558 | 35736868 | 11 |  |
| Ax1-11 | 9 | 3000351 | 3014078 | 7 |  |
| Ax1-11 | 9 | 3024426 | 3034834 | 5 |  |
| Ax1-11 | 15 | 101150866 | 101151174 | 14 |  |
| Ax1-14 | 2 | 98502403 | 98507339 | 4 |  |
| Ax1-14 | 12 | 7921237 | 7921438 | 8 | AK146888 |
| Ax1-14 | X | 143932298 | 143932828 | 12 | Dcx |
| Ax1-16 | 9 | 3000902 | 3004077 | 3 |  |
| Ax1-16 | 9 | 77355602 | 77355808 | 3 |  |
| Ax1-16 | X | 100516732 | 100525473 | 45 |  |
| Ax1-18 | 12 | 56243747 | 56243968 | 4 |  |
| Ax1-18 | 12 | 56318811 | 56319071 | 10 |  |
| Ax1-18 | 12 | 67829660 | 67829899 | 6 |  |
| Ax1-18 | X | 100516732 | 100525474 | 60 |  |
| Ax1-23 | 2 | 98507251 | 98507281 | 3 |  |
| Ax1-23 | 9 | 3003341 | 3018499 | 3 |  |
| Ax1-35 | 15 | 50590987 | 50591387 | 5 |  |
| Ax2-4 | 2 | 98506401 | 98507270 | 11 |  |
| Ax2-4 | 3 | 97567848 | 97567848 | 13 | Chd1l |
| Ax2-4 | 5 | 104683036 | 104683036 | 12 |  |
| Ax2-4 | 9 | 3000533 | 3017095 | 5 |  |
| Ax2-6 | 4 | 108996772 | 108997136 | 7 |  |
| Ax2-11 | 1 | 95594871 | 95595225 | 3 | St8sia4 |
| Ax2-11 | X | 100516717 | 100516717 | 10 | Dgat2l6 |
| Ax2-16 | 2 | 98506404 | 98507324 | 4 |  |
| Ax2-16 | 3 | 126629800 | 126629800 | 15 | Camk2d |
| Ax2-16 | 9 | 3000531 | 3014067 | 3 |  |
| Ax2-16 | 16 | 85940412 | 85940466 | 3 |  |
| Ax2-20 | 11 | 96209105 | 96209105 | 8 |  |
| Ax2-24 | 4 | 93684035 | 93684178 | 4 |  |
| Ax2-26 | 2 | 98502408 | 98507283 | 5 |  |
| Ax2-26 | 3 | 88776847 | 88777275 | 8 | Gon4l |
| Ax2-26 | 3 | 117503426 | 117503817 | 6 |  |
| Ax2-26 | 9 | 3000474 | 3017976 | 3 |  |
| Ax2-26 | 9 | 72125875 | 72125963 | 4 |  |
| Ax2-27 | 2 | 98502844 | 98507285 | 4 |  |
| Ax2-27 | 17 | 28731783 | 28731998 | 6 | Mapk14 |
| Ax2-30 | 2 | 98502394 | 98507363 | 29 |  |
| Ax2-30 | 2 | 146134656 | 146134990 | 13 | 4930529M08Rik |
| Ax2-30 | 7 | 79842317 | 79842317 | 15 | Anpep |
| Ax2-30 | 8 | 66526737 | 66526737 | 16 | AK009167 |
| Ax2-30 | 9 | 3000478 | 3020221 | 23 |  |
| Ax2-30 | 9 | 3023512 | 3032852 | 10 |  |
| Ax2-30 | 19 | 58931230 | 58931230 | 13 | Hspa12a |
| Ax2-30 | X | 100516717 | 100516717 | 10 | Dgat2l6 |
| Ax2-34 | 2 | 98502400 | 98507273 | 6 |  |
| Ax2-34 | 8 | 75425873 | 75426388 | 8 |  |
| Ax2-34 | 9 | 3000345 | 3014062 | 5 |  |
| Ax2-34 | 9 | 107928168 | 107928168 | 8 | AK014951 |
| Ax2-39 | 2 | 100796359 | 100796568 | 5 |  |
| Ax2-39 | X | 99161831 | 99162249 | 6 |  |
| Ax2-39 | X | 99570928 | 99571034 | 4 | M55023 |
| Ax2-48 | 2 | 98502810 | 98507283 | 6 |  |
| Ax2-48 | 7 | 19714217 | 19714426 | 8 | Tomm40 |
| Ax2-48 | 9 | 3000924 | 3014061 | 3 |  |
| Ax2-48 | 10 | 23250911 | 23251078 | 7 | Eya4 |
| Ax2-48 | 16 | 77574612 | 77574902 | 5 | 2810055G20Rik |
| Ax2-48 | X | 100516734 | 100525462 | 61 |  |
