# Supplementary material for: Genetic Heterogeneity of Induced Pluripotent Stem Cells: Results from 24 Clones Derived from a Single C57BL/6 Mouse
Source: PLoS One. 2015 Mar 23;10(3):e0120585. doi: 10.1371/journal.pone.0120585 (PMC4370741; doi:10.1371/journal.pone.0120585)
Supplement: S6 Table — (DOCX) [file pone.0120585.s006.docx]

**Table S6.** Common OSK lentiviral integration sites

| **Clone** | **Chromosome** | **Start** | **End** | **Supporting Reads** | **Gene location** |
| --- | --- | --- | --- | --- | --- |
| Ax2-11 | X | 100516717 | 100516717 | 10 | Dgat2l6 |
| Ax2-30 | X | 100516717 | 100516717 | 10 | Dgat2l6 |
