# Supplementary material for: Genetic Heterogeneity of Induced Pluripotent Stem Cells: Results from 24 Clones Derived from a Single C57BL/6 Mouse
Source: PLoS One. 2015 Mar 23;10(3):e0120585. doi: 10.1371/journal.pone.0120585 (PMC4370741; doi:10.1371/journal.pone.0120585)
Supplement: S7 Table — (DOCX) [file pone.0120585.s007.docx]

**Table S7.** Integration “hotspots”.

| **Clone** | **Chromosome** | **Start** | **End** | **Supporting Reads** |
| --- | --- | --- | --- | --- |
| Ax1-2 | 2 | 98506736 | 98507278 | 4 |
| Ax1-3 | 2 | 98502410 | 98507353 | 9 |
| Ax1-5 | 2 | 98502397 | 98507406 | 40 |
| Ax1-7 | 2 | 98506703 | 98507261 | 3 |
| Ax1-11 | 2 | 98502678 | 98507455 | 14 |
| Ax1-14 | 2 | 98502403 | 98507339 | 4 |
| Ax1-23 | 2 | 98507251 | 98507281 | 3 |
| Ax2-4 | 2 | 98506401 | 98507270 | 11 |
| Ax2-16 | 2 | 98506404 | 98507324 | 4 |
| Ax2-26 | 2 | 98502408 | 98507283 | 5 |
| Ax2-27 | 2 | 98502844 | 98507285 | 4 |
| Ax2-30 | 2 | 98502394 | 98507363 | 29 |
| Ax2-34 | 2 | 98502400 | 98507273 | 6 |
| Ax2-48 | 2 | 98502810 | 98507283 | 6 |
| Ax1-3 | 9 | 3002047 | 3007568 | 3 |
| Ax1-3 | 9 | 3024074 | 3027195 | 6 |
| Ax1-5 | 9 | 3000297 | 3017965 | 25 |
| Ax1-5 | 9 | 3020843 | 3032855 | 18 |
| Ax1-11 | 9 | 3000351 | 3014078 | 7 |
| Ax1-11 | 9 | 3024426 | 3034834 | 5 |
| Ax1-16 | 9 | 3000902 | 3004077 | 3 |
| Ax1-23 | 9 | 3003341 | 3018499 | 3 |
| Ax2-4 | 9 | 3000533 | 3017095 | 5 |
| Ax2-16 | 9 | 3000531 | 3014067 | 3 |
| Ax2-26 | 9 | 3000474 | 3017976 | 3 |
| Ax2-30 | 9 | 3000478 | 3020221 | 23 |
| Ax2-30 | 9 | 3023512 | 3032852 | 10 |
| Ax2-34 | 9 | 3000345 | 3014062 | 5 |
| Ax2-48 | 9 | 3000924 | 3014061 | 3 |
| Ax2-11 | X | 100516717 | 100516717 | 10 |
| Ax2-30 | X | 100516717 | 100516717 | 85 |
| Ax1-16 | X | 100516732 | 100525473 | 45 |
| Ax1-18 | X | 100516732 | 100525474 | 60 |
| Ax2-48 | X | 100516734 | 100525462 | 61 |
